# Early administration of SGLT2 inhibitors in hospitalized patients: A practical guidance from the current evidence

**DOI:** 10.1002/ehf2.15293

**Published:** 2025-04-17

**Authors:** Ruggero Mazzotta, Manuel Garofalo, Samuele Salvi, Matteo Orlandi, Gianluca Marcaccini, Pietro Susini, Luca Checchi, Alberto Palazzuoli, Carlo Di Mario, Maurizio Pieroni, Matteo Beltrami

**Affiliations:** ^1^ Careggi University Hospital Florence Italy; ^2^ Department of Experimental and Clinical Medicine University of Florence Florence Italy; ^3^ Department of Medicine, Surgery and Neuroscience University of Siena Siena Italy; ^4^ Arrhythmia and Electrophysiology Unit Careggi University Hospital Florence Italy; ^5^ Cardiovascular Diseases Unit, Cardio‐Thoracic and Vascular Department Le Scotte Hospital University of Siena Siena Italy

**Keywords:** Acute heart failure, Heart failure, SGLT2 inhibitors, Worsening heart failure

## Abstract

Sodium‐glucose cotransporter‐2 (SGLT2) inhibitors represent one of the main cornerstones of heart failure treatment. Nevertheless, while the cardiovascular beneficial effects of these drugs have been clearly demonstrated by several clinical trials, in clinical practice, it remains challenging to identify the appropriate timing to start SGLT2 inhibitors. The potential risk of side effects, like genito‐urinary infections and interaction with other drugs, may often lead to delay the prescription of these drugs in the acute setting. However, several studies have demonstrated the safety and the prognostic impact of SGLT2 inhibitors in the hospitalized patient, suggesting that treatment initiation during hospitalization or early post‐discharge may represent an ideal therapeutic option. In this review, we discuss the main trials on early administration of SGLT2 inhibitors in acute heart failure supporting early introduction of SGLT2 inhibitors to optimize heart failure treatment. The efficacy and safety of these drugs in patients with acute myocardial infarction are also discussed. Based on the review of existing evidences, a practical flowchart on early administration of SGLT2 inhibitors in the acute setting is proposed.

## Graphical abstract

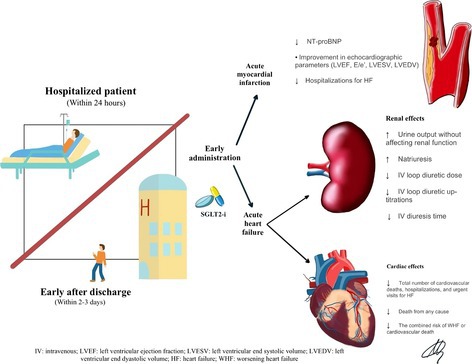

## 1 Introduction

Several trials have consistently demonstrated the beneficial effects of sodium‐glucose cotransporter 2 (SGLT2) inhibitors on mortality and re‐hospitalization in heart failure (HF) patients. These evidences led to update clinical guidelines and to include this class of drugs among first‐line therapies for chronic heart failure (CHF) treatment across the complete spectrum of left ventricular ejection fraction (LVEF).^1^, ^2^, ^3^, ^4^, ^5^, ^6^, ^7^ Guidelines, however, remain largely disattended with only a minority of patients on optimal HF medical therapy.^8^, ^9^ Most individuals with CHF develop clinical and haemodynamic deterioration during episodes of acute heart failure (AHF), a severe phase of this dynamic condition burdened by high in‐hospital and post‐discharge 1‐year mortality. STRONG (Safety, tolerability and efficacy of up‐titration of guideline‐directed medical therapies for acute heart failure) and COACH (Trial of an Intervention to Improve Acute Heart Failure Outcomes) trials clearly demonstrated a reduction in adverse events associated with the simultaneous insertion of the four pillars of HF treatment.^10^, ^11^

In‐hospital initiation of HF therapies is associated with greater prognostic benefit and long‐term adherence.^12^, ^13^, ^14^ Accordingly, concerns about the safety and efficacy of initiating SGLT2 inhibitors during hospitalization for acutely decompensated heart failure (ADHF)—including their renal effects—are minimal, even in patients with pre‐existing hypotension or chronic kidney disease (CKD).^11^

**Figure 1:**
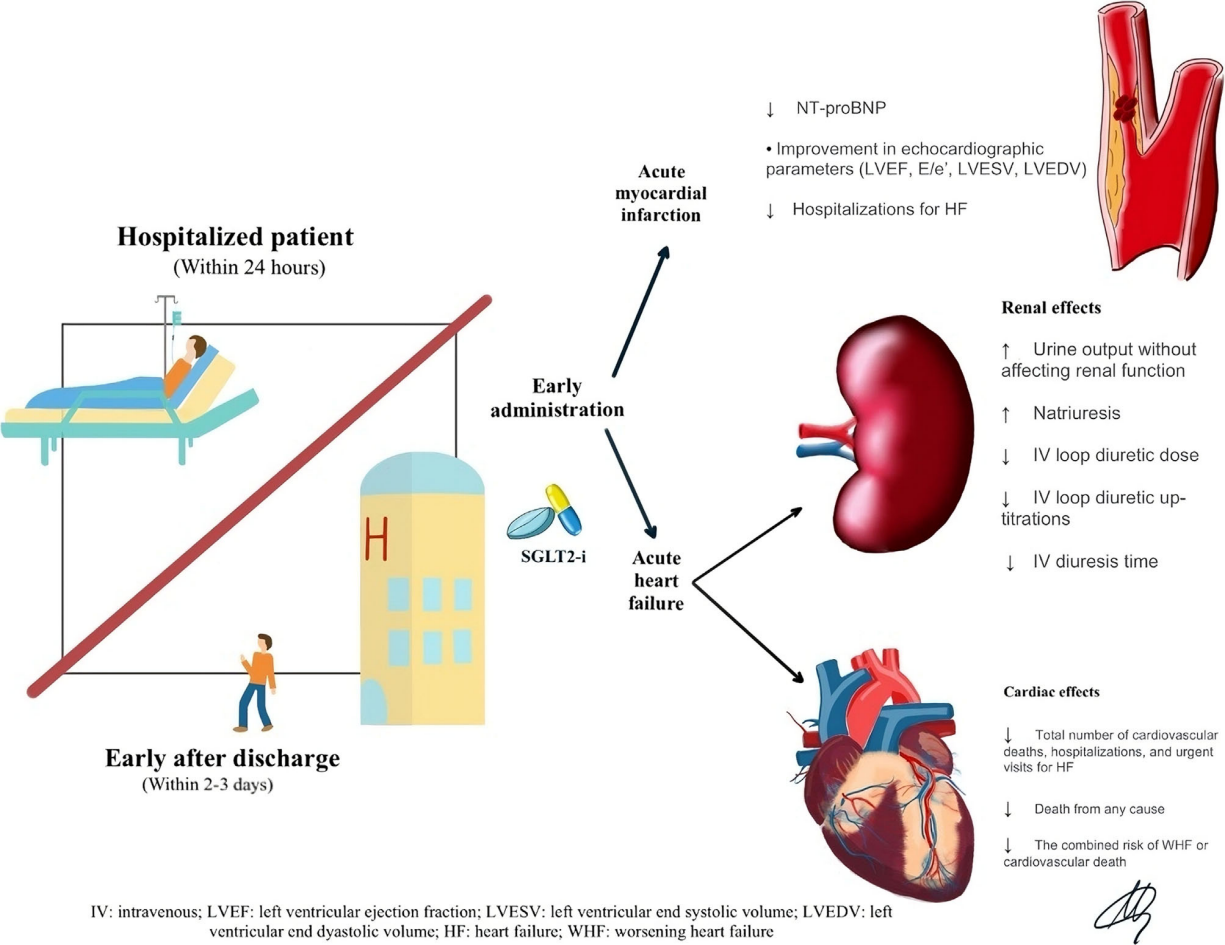
Main benefits of early administration of SGLT2 inhibitors during hospitalization or shortly after discharge, for both acute heart failure and acute myocardial infarction.

This review aims to evaluate key clinical trials on this topic, focusing on the early in‐hospital or post‐discharge administration of SGLT2 inhibitors in two specific settings: AHF and acute myocardial infarction (AMI) (*Figure* *1*). We will also address issues on tolerability and safety of early administration of these drugs in clinical practice, offering a practical flowchart on how to start SGLT2 inhibitors in acute settings.

## 2 Efficacy in acute heart failure

The rapid divergence of survival curves with SGLT2 inhibitors in landmark trials on CHF, such as DAPA‐HF (Dapagliflozin in Patients with Heart Failure and Reduced Ejection Fraction)^15^ and EMPEROR‐Reduced (Cardiovascular and Renal Outcomes with Empagliflozin in Heart Failure)^1^ highlighted their early clinical benefits. The DAPA‐HF showed a significative separation of the Kaplan Meier curves after 28 days from randomization (HR, 0.51; 95% CI, 0.28 to 0.94; *P* = 0.03), while in the EMPEROR‐Reduced the curves separated 12 days after randomization (HR, 0.76; 95% CI, 0.67 to 0.87; *P* < 0.0001). Recent studies on the time‐to‐benefit of SGLT2 inhibitors have shown a rapid and clinically significant reduction in the risk of cardiovascular death or worsening heart failure (WHF).^16^ These findings underscored the broad benefits of SGLT2 inhibitors, further supporting their use in the AHF setting. The SOLOIST‐WHF (Effect of Sotagliflozin on Cardiovascular Events in Participants With Type 2 Diabetes Post Worsening Heart Failure) trial examined the role of gliflozins in hospitalized or recently discharged AHF patients (within 3 days from discharge). This multicentre, double‐blind, randomized trial evaluated 1222 patients with type 2 diabetes mellitus (T2DM) recently hospitalized for WHF, with a median follow‐up of 9 months. Sotagliflozin (a combined sodium‐glucose cotransporter‐1 and SGLT2 inhibitor) or placebo were administered in 48.8% of patients before discharge and in 51.2% of patients soon after discharge, starting with a median of 2 days. Event rates per 100 patient‐years were lower in the sotagliflozin group than in the placebo group (51.0 vs. 76.3; HR, 0.67; 95% CI, 0.52–0.85; *P* < 0.001). There was also a non‐significant trend towards lower all‐cause mortality (10.6% vs. 12.5%; HR, 0.84; 95% CI, 0.58–1.22) and cardiovascular mortality (13.5% vs. 16.3%; HR, 0.82; 95% CI, 0.59–1.14).^15^ In a post‐hoc analysis of the SOLOIST‐WHF study, starting sotagliflozin prior to discharge was found to significantly reduce the combined endpoint at 90 days (HR, 0.54; 95% CI, 0.35 to 0.82; *P* = 0.004) and at 30 days (HR, 0.49; 95% CI, 0.27 to 0.91; *P* = 0.023) and all‐cause mortality at 90 days (HR, 0.39; 95% CI, 0.17 to 0.88; *P* = 0.024). This significance remained even after adjustment for sex, age, estimated glomerular filtration rate (eGFR), N‐terminal pro‐B‐type natriuretic peptide (NT‐proBNP) levels, LVEF and mineralocorticoid receptor antagonists (MRAs) use.^17^ In the EMPULSE (Empagliflozin in Patients Hospitalized With Acute Heart Failure Who Have Been Stabilized) trial 530 patients with de novo AHF or chronic decompensated HF, regardless of LVEF, were randomized as soon as they became clinically stable (median time from randomization, 3 days). Early in‐hospital administration of empagliflozin resulted in a statistically significant reduction in the primary combined endpoint at 90 days (hierarchical composite of death from any cause, number of HF events, time to first HF event, difference of 5 or more points in the change from baseline in the Kansas City Cardiomyopathy Questionnaire Total Symptom Score) with a stratified win ratio of 1.36 (*P* = 0.005). The effect was consistent across a broad spectrum of patient characteristics, including de novo AHF and chronic decompensated HF, the presence or absence of diabetes, and all categories of LVEF.^18^, ^19^ As residual congestion remains a key predictor of poor outcome, its recognition and management are primary goals during hospitalization.^20^, ^21^, ^22^, ^23^ A post‐hoc analysis of the EMPULSE trial examined decongestion‐related endpoints—including weight loss (WL), WL adjusted for mean daily loop diuretic dose (WL‐adjusted), and clinical congestion score—at 15, 30, and 90 days, assessing their impact on short‐term prognosis. Patients treated with empagliflozin experienced significantly greater reductions in all these markers. Notably, a more pronounced weight loss by day 15 was associated with a significantly higher likelihood of clinical benefit at 90 days.^24^ Ferreira et al. have evaluated changing in clinical events and treatment effects according to time from admission to randomization. Patients randomized later (3–5 days after admission) had a higher risk of clinical events than patients randomized earlier (1–2 days). Second, the treatment effect favoured empagliflozin versus placebo in patients randomized later (3–5 days; WR, 1.69; 95% CI, 1.26 to 2.25) but was attenuated in patients randomized earlier (1–2 days; WR, 1.04; 95% CI, 0.74 to 1.44) (p for interaction = 0.029).^25^ These findings may be explained by the fact that patients randomized earlier achieved clinical stabilization more quickly, requiring lower cumulative diuretic doses, and thus represented a lower‐risk group. Moreover, in a subsequent analysis of the EMPULSE, the primary outcome was not influenced by baseline MRAs use.^26^

The EMPA‐RESPONSE‐AHF (Effects of Empagliflozin on Clinical Outcomes in Patients with Acute Decompensated Heart Failure) trial enrolled 80 ADHF patients (47% of whom with de novo HF) randomized to either empagliflozin 10 mg/day or placebo within 24 h of admission. Empagliflozin reduced the combined endpoint of in‐hospital WHF, rehospitalization for HF or death at 60 days compared with placebo [4 (10%) vs. 13 (33%); *P* = 0.014].^27^ In a substudy of the DELIVER (Dapagliflozin in Heart Failure with Mildly Reduced or Preserved Ejection Fraction) trial including patients recently hospitalized for heart failure with mildy reduced ejection fraction (HFmrEF) or heart failure with preserved ejection fraction (HFpEF), out of intravenous administration treatment and in stable conditions, dapagliflozin reduced the primary endpoint of WHF or cardiovascular death by 22% in recently hospitalized patients and 18% in patients without a recent hospitalization, while the incidence of adverse events was similar in the two groups.^4^ Relative reductions in the primary outcome were consistent in patients with and without history of recent HF hospitalization.^28^, ^29^

The DAPA‐RESPONSE‐AHF (The Clinical Outcomes of Dapagliflozin in Patients with Acute Heart Failure) trial included 87 AHF patients within 24 h of hospital admission. Dapagliflozin significantly improved the visual analogue scale dyspnoea score compared with placebo and reduced 30‐day rehospitalization rates. However, it did not impact WHF incidence or mortality rates.^30^

A recent meta‐analysis confirmed that early administration of SGLT2 inhibitors in the AHF setting leads to a significant risk reduction of 62% in HF events, defined as a recurrent HF hospitalization/emergency department admissions, urgent care visits, or the need for intensification of management during outpatient follow‐up consultations.^31^ The main results of randomized controlled trials (RCTs) investigating SGLT2 inhibitors in the acute setting are summarized in *Table* *1*.

**Table 1:** Design and main results of randomized controlled trials in the acute heart failure setting.

RCT (year)	Patients (n)	Timing of SGLT2‐inhibitor administration	Type and dose of SGLT2‐inhibitor	Primary endpoint	Results
EMPA‐RESPONSE‐AHF (2020)	Patients with AHF (80).	Within 24 h of hospitalization.	Empagliflozin 10 mg once daily.	Change in VAS dyspnoea score, diuretic response, change in NT‐proBNP, and length of stay.	• No difference was observed in the primary endpoint between empagliflozin and placebo. • Empagliflozin reduced a combined endpoint of in‐hospital WHF, rehospitalization for HF or death at 60 days compared with placebo [4 (10%) vs. 13 (33%); *P* = 0.014].
SOLOIST‐WHF (2020)	Patients with T2DM who were recently hospitalized for WHF and received treatment with intravenous diuretics (1222).	Before discharge in 48.8% and a median of 2 days after discharge in 51.2% of patients.	Sotagliflozin 200 mg once daily (with a dose increase to 400 mg, depending on side effects).	Total number of deaths from cardiovascular causes and hospitalizations and urgent visits for HF (first and subsequent events).	• The number of events per 100 patient‐years of primary endpoint events was lower in the sotagliflozin group versus placebo group (51.0 vs. 76.3; HR, 0.67; 95% CI, 0.52 to 0.85; *P* < 0.001). • The rate of death from cardiovascular causes was 10.6 in the sotagliflozin group and 12.5 in the placebo group (HR, 0.84; 95% CI, 0.58 to 1.22); the rate of death from any cause was 13.5 in the sotagliflozin group and 16.3 in the placebo group (HR, 0.82; 95% CI, 0.59 to 1.14).
EMPULSE (2022)	Patients with de novo AHF or chronic decompensated HF (530).	Median time from hospital admission to randomization was 3 days.	Empagliflozin 10 mg once daily.	Hierarchical composite of death from any cause, number of HF events and time to first HF event, or a 5 point or greater difference in change from baseline in the KCCQ Total Symptom Score at 90 days.	More patients treated with empagliflozin had clinical benefit compared with placebo (stratified win ratio, 1.36; 95% CI, 1.09 to 1.68; *P* = 0.0054).
EMPAG‐HF (2022)	Patients with ADHF.^32^	Within 12 h of hospitalization.	Empagliflozin 25 mg once daily.	Cumulative urine output over 5 days.	Addition of empagliflozin resulted in a 25% increase in cumulative urine output over 5 days (median 10.8 vs. 8.7 L in placebo; group difference estimation 2.2 L; 95% CI, 8.4 to 3.6; *P* = 0.003).
DAPA‐RESPONSE‐AHF (2023)	Patients with AHF (87).	Within 24 h of hospitalization.	Dapagliflozin 10 mg once daily.	Difference AUC of VAS dyspnoea score over the first 4 days.	• Dapagliflozin significantly reduced the AUC of VAS dyspnoea score compared with placebo (3192.2 ± 1631.9 mm × h vs. 4713.1 ± 1714.9 mm × h; *P* < 0.001). • The relative change of NT‐proBNP compared with its baseline was larger with dapagliflozin (−34.89% vs. −10.085%; *P* = 0.001). • Higher cumulative urine output was found at day 4 (18 600 mL in dapagliflozin vs. 13 700 in placebo; *P* = 0.031). • Dapagliflozin decreased rehospitalization rates within 30 days after discharge.
DICTATE‐AHF (2024)	Patients with AHF and current or planned treatment with intravenous diuretics (240).	Within 24 h of hospitalization.	Dapagliflozin 10 mg once daily.	Diuretic efficiency, expressed as cumulative weight change per cumulative loop diuretic dose.	For diuretic efficiency, no difference between dapagliflozin and usual care was observed (OR, 0.65; 95% CI, 0.41 to 1.02; *P* = 0.06).

ADHF, acutely decompensated heart failure; AHF, acute heart failure; AUC, area under the curve; HF, heart failure; KCCQ, Kansas City Cardiomyopathy Questionnaire; NT‐proBNP, N‐terminal prohormone of brain natriuretic peptide; T2DM, type 2 diabetes mellitus; VAS, visual analogue scale; WHF, worsening heart failure.

## 3 Efficacy in acute myocardial infarction

Unlike the AHF setting, the three major trials evaluating the prognostic impact of SGLT2 inhibitors in AMI yielded conflicting results (*Table* *2*). In the EMMY (Empagliflozin in Acute Myocardial Infarction) multicentre, double‐blind, randomized trial, 476 patients with AMI were randomly assigned to empagliflozin or placebo within 72 h of percutaneous coronary intervention. The primary endpoint was the NT‐proBNP change over 26 weeks and secondary outcomes included changes in echocardiographic parameters. Empagliflozin was associated with a greater NT‐proBNP reduction over 26 weeks, accompanied by a significant decrease in left ventricular volumes and simultaneous improvement in LVEF and mean E/e'.^33^ These promising results, which focused on mechanistic, non‐clinical surrogate endpoints, were followed by two larger trials that failed to confirm a clear prognostic benefit in this setting. In the DAPA‐MI (Dapagliflozin in Patients without Diabetes Mellitus with Acute Myocardial Infarction) trial, 4017 patients without prior diabetes or CHF, presenting with AMI and impaired LVEF, were randomly assigned to placebo or dapagliflozin, with the first dose administrated during hospitalization or within 10 days. At a mean follow‐up of approximately 1 year dapagliflozin showed significant improvement in the cardiometabolic status but had no impact on the primary endpoint, a composite of cardiovascular death or HF hospitalization.^34^ The EMPACT‐MI (Effect of Empagliflozin on Heart Failure Outcomes After Acute Myocardial Infarction) trial randomized 6522 AMI patients at high risk of developing HF (aged 65 and above, with newly developed LVEF of less than 35%, history of previous myocardial infarction, atrial fibrillation, T2DM, and other factors associated with increased HF risk) to the administration of empagliflozin or placebo within 14 days after hospital admission. The composite endpoint of first hospitalization for HF and all‐cause death was similar between the two groups, occurring in 267 patients (8.2%) in the empagliflozin group and 298 patients (9.1%) in the placebo group (HR, 0.90; 95% CI, 0.76 to 1.06; *P* = 0.21).^35^ However, a prespecified secondary analysis showed that the risk of first HF hospitalization was lower in patients randomized to empagliflozin compared with placebo (118 vs. 153 events; HR, 0.77; 95% CI, 0.60 to 0.98; *P* = 0.031). This analysis also found a significant reduction in total HF hospitalizations (148 vs. 207 events; RR 0.67; 95% CI, 0.51–0.89; *P* = 0.006). The risk of HF hospitalization or death attributable to HF was significantly reduced in the empagliflozin group.^36^ These results were confirmed by a recent large meta‐analysis: SGLT2 inhibitors initiation after AMI was safe and was associated with a reduced risk of HF hospitalizations, but not with all‐cause mortality..^37^

**Table 2:** Design and main findings of randomized controlled trials in the acute myocardial infarction setting.

RCT	Patients (*n*)	Timing of SGLT2‐inhibitor administration	Type and dose of SGLT2‐inhibitor	Primary endpoint	Results
EMMY(2022)	Patients with AMI (476).	Within 72 h after a PCI.	Empagliflozin 10 mg once daily.	NT‐proBNP change over 26 weeks.	NT‐proBNP reduction was significantly greater in the empagliflozin group, compared with placebo (*P* = 0.026).
DAPA‐MI (2023)	Patients with AMI and impaired left ventricular systolic function (4017).	During the hospitalization for the index AMI event or within 10 days from index AMI.	Dapagliflozin 10 mg once daily.	Hierarchical composite of death, hospitalization for HF, nonfatal AMI, atrial fibrillation/flutter, T2DM, New York Heart Association Functional Classification at the last visit, and body weight decrease of 5% or greater at the last visit.	The analysis of the primary hierarchical composite outcome resulted in significantly more wins for dapagliflozin than for placebo (win ratio, 1.34; 95% CI, 1.20 to 1.50; *P* < 0.001).
EMPACT‐MI (2024)	Patients who had been hospitalized for AMI and were at risk for HF (6522).	Within 14 days after admission.	Empagliflozin 10 mg once daily.	Composite of hospitalization for HF or death from any cause as assessed in a time‐to‐first‐event analysis.	Incidence rates of the primary endpoint were 5.9 per 100 patient‐years in the empagliflozin group and 6.6 events per 100 patient‐years in the placebo group (HR, 0.90; 95% CI, 0.76 to 1.06; *P* = 0.21).

AMI, acute myocardial infarction; HF, heart failure; NT‐proBNP, N‐terminal prohormone of brain natriuretic peptide; PCI, percutaneous coronary intervention; T2DM, type 2 diabetes mellitus.

Furthermore, the EMBODY trial evaluated the effect of empagliflozin on cardiac sympathetic and parasympathetic activity assessing heart rate variability and heart rate turbulence. Treatment with empagliflozin lead to significant improvement in both cardiac sympathetic and parasympathetic nerve activities in patients with T2DM and AMI.^36^

These data suggest that empagliflozin could potentially have a role even in AMI patients at risk of developing HF. The lack of prognostic and clinical benefits may have multiple explanations. First of all, differences in patients' baseline clinical and echocardiographic characteristics (i.e. the degree of left ventricular adverse remodelling and systolic dysfunction) could have resulted in enrolling individuals across a wide range of HF stages. Secondly, despite the decision to increase the number of patients enrolled, the event rate remained very low. The trial was conducted during the COVID‐19 pandemic, which likely influenced the number of HF hospitalizations, a key driver of treatment benefit in AHF trials. In the AMI setting, early mortality is often driven by recurrent ischemic or arrhythmic events, which are unlikely to be significantly affected by empagliflozin. Additionally, factors such as coronary artery disease severity, myocardial scarring, and hibernating tissue extent—each influencing revascularization strategies—may introduce further bias.

## 4 Beneficial effects on renal function and kalemia

### 4.1 A close look inside kidney protection

Renin angiotensin system (RAAS) inhibitors, ARNI and SGLT2 inhibitors significantly impact the renal function due to changes in renal physiology. SGLT2 inhibitors modulate renal function by resetting the renal function curve and influencing the relationship between intraglomerular hydrostatic pressure and natriuresis. This effect is mediated through tubuloglomerular feedback and by counteracting the sympathetic activity‐induced alterations in the afferent and efferent glomerular arterioles. These effects modify the physiological filtration fraction, have different baroceptorial and chemotactic repercussion on the macula densa, and may impact the tubular function.^38^, ^39^, ^40^ The acute reduction in intraglomerular pressures reduces both the left ventricular afterload and preload and decreases blood pressure and arterial stiffness. These mechanisms could explain the initial decrease (early dip) in eGFR,^41^, ^42^, ^43^ a pharmacodynamic effect not associated with long‐term worsening of renal function or with other renal adverse events. Patients with diabetes tend to have a more pronounced acute drop in eGFR, potentially reflecting the initial higher intraglomerular pressures. The slight, transient drop in eGFR following SGLT2 inhibitor initiation should not be interpreted as renal function deterioration but rather as a nephroprotective glomerular response to cotransporter blockade. Therefore, it should not warrant treatment discontinuation.^44^, ^45^, ^46^, ^47^, ^48^ By increasing diuresis and urinary output, they could be useful as an adjuvant therapy to achieve a greater degree of pre‐discharge net fluid loss without worsening renal function.^49^ The EMPAG‐HF (Effects of Early Empagliflozin Initiation on Diuresis and Kidney Function in Patients With Acute Decompensated Heart Failure) trial demonstrated that, in ADHF patients randomized to empagliflozin or placebo within 12 h of hospitalization, the early addition of empagliflozin to standard diuretic therapy was associated with a therapeutic benefit without kidney safety issues. Empagliflozin addition to standard medical treatment resulted in a 25% increase in cumulative urine output over 5 days (median 10.8 vs. 8.7 L mL in placebo; group difference estimation 2.2 L; *P* = 0.003) without affecting renal function (eGFR, 51 ± 19 vs. 54 ± 17 mL/min per 1.73 m^2^), total urinary proteins (492 ± 845 vs. 503 ± 847 mg/g creatinine; *P* = 0.975) and urinary α1‐microglobulin (55.4 ± 38.6 vs. 31.3 ± 33.6 mg/g creatinine; *P* = 0.066). There was no significant difference in the change of eGFR between the two groups at different time points after randomization.^50^

### 4.2 Decongestional properties

The addition of SGLT2 inhibitors to conventional diuretic therapy resulted in an increased urinary output and a decreased mean daily doses of loop diuretics without affecting the renal function.^51^ In the EMPA‐RESPONSE‐AHF trial, urinary output until day 4 was significantly greater with empagliflozin versus placebo without increasing the incidence of renal adverse events.^27^ The DICTATE‐AHF (Efficacy and safety of dapagliflozin in acute heart failure) trial demonstrated that in hypervolaemic AHF patients, dapagliflozin was associated with a reduction in loop diuretic doses (and fewer intravenous diuretic up‐titrations to achieve equivalent weight loss as usual care), even if not associated with a statistically significant reduction in weight‐based diuretic efficiency.^15^, ^52^ The DAPA‐RESPONSE AHF trial showed an increase in cumulative urine output and diuretic efficiency in dapagliflozin group compared with placebo group at day 4 with a similar incidence of worsening renal function.^30^ In the EMPULSE trial, acute kidney injury occurred in 7.7% of patients receiving empagliflozin compared with 12.1% of those in the placebo group. The change in creatinine levels between baseline and the last value on treatment was similar between the two groups. The authors also noted that the slight decline in eGFR observed at the beginning of treatment was no longer present at the 90‐day follow‐up.^18^ No safety concerns were identified in the SOLOIST‐WHF trial, where the incidence of acute kidney injury was comparable between patients treated with sotagliflozin and those receiving placebo.

The DAPA‐RESIST (Dapagliflozin vs. Metolazone in Heart Failure Resistant to Loop Diuretics) trial randomized 61 ADHF patients with diuretic resistance to receive dapagliflozin 10 mg daily or metolazone 5 or 10 mg daily for up to three consecutive days with a median follow‐up of 90 days. Even though dapagliflozin was not more effective than metolazone in lowering congestion grade, it was associated with a lower reduction in serum sodium and potassium concentrations and a smaller increase in blood urea nitrogen and serum creatinine.^53^ The early use of SGLT2 inhibitors is associated with a greater degree of decongestion and an excellent safety profile when added to loop diuretics or thiazide‐like diuretics.

In clinical practice, close monitoring of renal function is essential. Initiating treatment with dapagliflozin is not recommended for patients with an eGFR below 25 mL/min/1.73 m^2^, nor with empagliflozin if eGFR is below 20 mL/min/1.73 m^2^.

### 4.3 Effect on potassium level

Hyperkalaemia represents a frequent challenge in HF patients and is influenced by neurohormonal activation related to HF, coexisting conditions such as CKD and diabetes, and the concomitant use of other pharmacological HF pillars such as ARNI and MRAs. The presence of hyperkalaemia can severely limit either the initiation and rapid up‐titration of HF treatment. Even if in a chronic outpatient setting, promising findings from a pooled analysis of the EMPEROR‐Reduced and EMPEROR‐Preserved (Empagliflozin in Heart Failure with a Preserved Ejection Fraction) trials showed that empagliflozin can reduce the composite endpoint of investigator‐reported hyperkalemia or the need for potassium binders, compared with placebo (6.5% vs. 7.7%; HR, 0.82; 95% CI, 0.71 to 0.95; *P* = 0.01). Empagliflozin also lowered the overall incidence of hyperkalaemia (6.1% vs. 7.2%, HR, 0.83; 95% CI, 0.71 to 0.97; *P* = 0.018), without significantly increasing the risk of hypokalaemia. These benefits were observed across various subgroups including different LVEFs, diabetes status, and MRAs use, with the effects being particularly pronounced in patients with lower eGFR.^54^ A recent meta‐analysis involving patients with T2DM at high cardiovascular risk or with CKD revealed a significant reduction in the incidence of severe hyperkalaemia (defined as serum potassium ≥6.0 mmol/L) compared with placebo (HR, 0.84; 95% CI, 0.76 to 0.93). These results were consistent across a range of subgroups, including baseline kidney function and HF therapies (ARNI and RAAS inhibitors).^55^ A meta‐analysis of five clinical trials (11 123 patients already treated with MRAs) evaluating SGLT2 inhibitors versus placebo showed that the combination of SGLT2 inhibitors and MRAs significantly reduced the risk of hyperkalaemia (HR, 0.61; 95% CI, 0.50 to 0.75; *P* < 0.00001) compared with MRAs therapy alone.^56^ These findings highlight the potential role of SGLT2 inhibitors in preventing hyperkalaemia following the initiation or up‐titration of other heart failure therapies, thereby enhancing the tolerability of ARNI and MRAs treatment.^55^, ^57^, ^58^

## 5 Tolerability and safety

### 5.1 Hypotension

Since SGLT2 inhibitors promote osmotic diuresis and reduce intravascular volume, there have been concerns about the potential for symptomatic hypotension in patients after their initiation. A close monitoring of this potential side‐effect is crucial as hypotension can hinder the introduction and up‐tritation of HF medications. Moreover, hypotension can impair peripheral perfusion, heightening the risk of organ dysfunction, including acute renal failure.^59^ Despite initial concerns, major clinical trials demonstrated that SGLT2 inhibitors have modest or negligible lowering effect on blood pressure compared with placebo. In the EMPEROR‐Reduced (Effect of Empagliflozin on the Clinical Stability of Patients With Heart Failure and a Reduced Ejection Fraction) trial, patients treated with empagliflozin showed a reduction in systolic blood pressure (SBP) of −2.4 mmHg versus −1.7 mmHg on placebo, with a clinically negligible absolute mean difference of just −0.7 mmHg and no significant differences in the development of symptomatic or asymptomatic hypotension.^1^ A secondary analysis of this trial revealed that the effects of empagliflozin on cardiovascular death or HF hospitalization were consistent across different SBP ranges. Nearly a quarter of the patients had a baseline SBP between 100 and 110 mmHg, yet they did not exhibit a higher risk of either symptomatic and asymptomatic hypotension compared with those with higher SBP. Importantly, the treatment benefit for the primary outcome remained consistent across all SBP groups.^60^ Similar findings were also observed in several major clinical trials.^14^, ^16^ Although SGLT2 inhibitors have a largely neutral effect on blood pressure, all major trials excluded patients with an SBP below 100 mmHg. Consequently, their use is generally not recommended below this cut‐off.^42^

### 5.2 Genital infections and euglycaemic ketoacidosis

The most common adverse effects of SGLT2 inhibitors, strictly connected with their glycosuric action, are both mycotic genital and urinary tract infections. Women are particularly vulnerable to these conditions, which typically manifest as mild infections but can, in rare cases, lead to severe complications including sepsis and death.^32^ Serious but uncommon events like pyelonephritis and Fournier's gangrene have also been reported.^61^ Interestingly, most major clinical trials found no significant difference in the incidence of genitourinary infections between SGLT2 inhibitors and placebo groups. A meta‐analysis of 72 smaller RCTs found no difference in urinary tract infection rates between SGLT2 inhibitors and control groups, but indicated an increased risk of genital infections (RR, 3.37; 95% CI, 2.89 to 3.93).^62^ Conversely, a more recent meta‐analysis of nine RCTs involving patients without T2DM showed an increased risk of both genitourinary and genital infections with SGLT2 inhibitors (OR, 1.33; 95% CI, 1.13 to 1.57).^63^

The infections are generally symptomatic and typically respond well to topical or single‐dose oral antifungal or antibiotic treatments, usually without requiring the discontinuation of SGLT2 inhibitors. Maintaining good genital hygiene can also reduce the incidence of these complications.

Another rare but potentially life‐threatening adverse effect of SGLT2 inhibitors is euglycaemic ketoacidosis. This condition is characterized by the accumulation of ketone bodies, primarily hydroxybutyric acid, and can develop despite normal blood glucose levels. Symptoms are often vague, including polyuria, abdominal pain, nausea or vomiting, and confusion.^64^

Beyond metabolic conditions, patients with more advanced heart failure and peripheral hypoperfusion, or those with a concurrent systemic infection leading to elevated lactate levels, are more susceptible to metabolic acidosis. Individuals with alcohol dependence, pancreatic disease, and elderly patients experiencing volume depletion are at heightened risk.^32^ Similar to euglycaemic ketoacidosis associated with metformin use, this adverse event can be triggered by prolonged fasting, acute illness, or surgical procedures.^42^ Given these risks, the 2022 European Society of Cardiology (ESC) guidelines advise discontinuing SGLT2 inhibitors for at least 3 days before intermediate‐ and high‐risk non‐cardiac surgeries.^65^

## 6 Timing of SGLT2 inhibitors early administration

**Figure 2:**
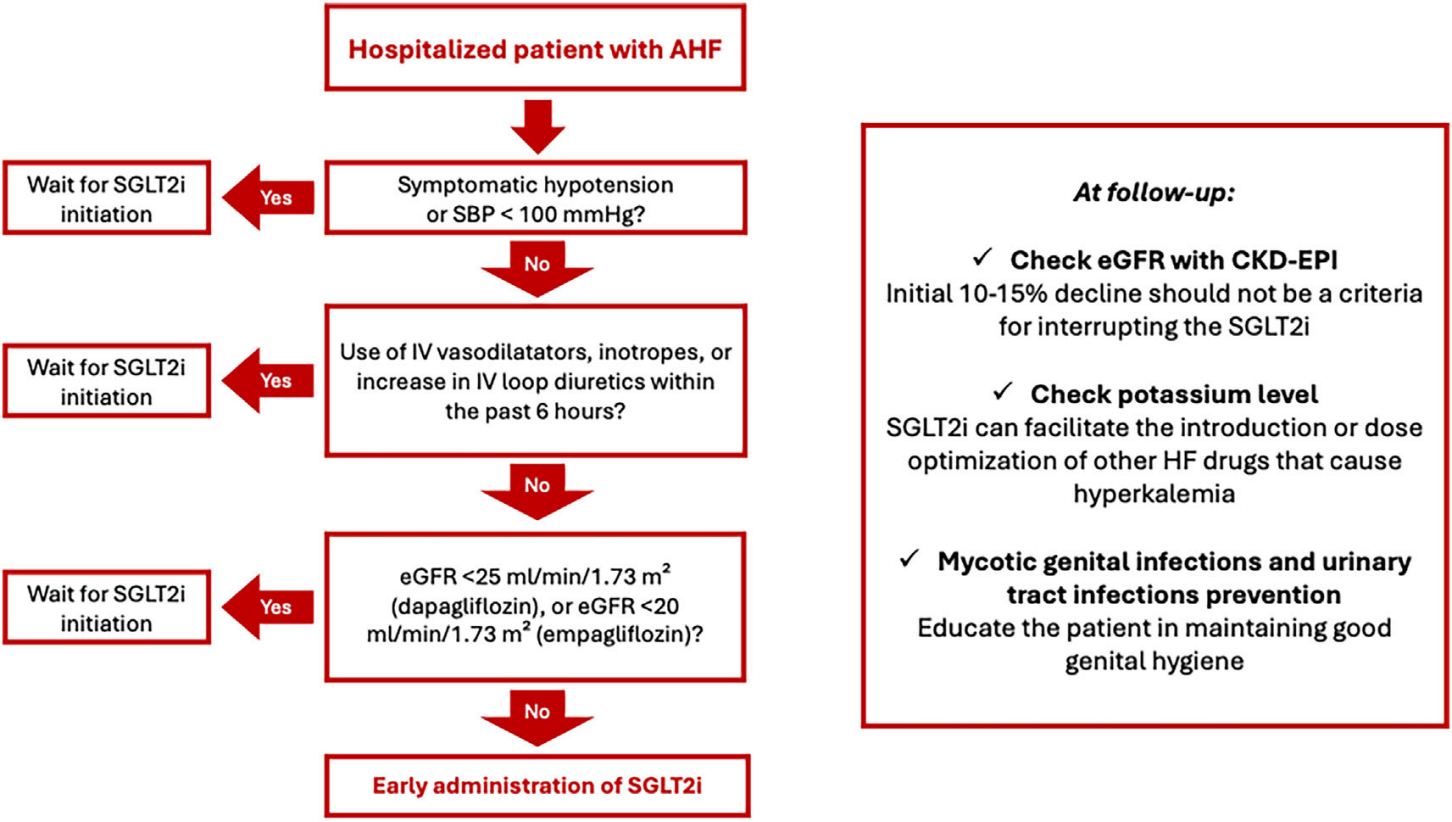
Practical flowchart for a rapid in‐hospital SGLT2 inhibitors initiation.

The 2023 focus update of ESC HF guidelines give a Class I recommendation to a rapid sequencing strategy and up‐titration of evidence‐based treatment before discharge and during early follow‐up visits after a HF hospitalization. The current approach recommends starting with low doses of all four drug classes, prioritizing their initiation over the up‐titration of any single drug class to its target dose. Combining current HF lifesaving drugs significantly improved the hard endpoints in the HF population.^7^, ^66^ STRONG‐HF trial showed the safety and efficacy of an approach based on starting and/or up‐titrating oral medical therapy for HF within 2 days before hospital discharge or early after discharge. Patients receiving early and rapid intensification of oral HF treatment with angiotensin converting enzyme (ACE) inhibitors or angiotensin receptor blockers (ARBs) or ARNI, beta‐blockers, and MRAs showed a significant better outcome without safety concerns.^10^ Although SGLT2 inhibitors were not evaluated in this trial, this recommendation also includes empagliflozin or dapagliflozin based on recent evidence.^3^, ^4^, ^18^ Their early initiation during hospitalization and continued use after discharge provide clinical benefits within less than 2 weeks. However, data on their long‐term effects remain limited.^18^, ^67^, ^68^ Although there is little evidence comparing the sequence of therapy initiation, particularly in patients with de novo heart failure with reduced ejection fraction (HFrEF), there is a growing emphasis on the systematic introduction of all four major therapy classes for HFrEF after stabilization, along with diuretics as needed to relieve and prevent congestion.^69^ Due to the protective vascular and hormonal actions of SGLT2 inhibitors, the use of this agent may be safely extended to patients with renal dysfunction and/or hyperkalaemia in the long‐term facilitating the initiation of other HF therapies. SGLT2 inhibitors can improve the tolerability of other foundational therapies, making proper sequencing essential for ensuring the safety of medications introduced later. However, the extensive application of multiple HF agents needs caution and a frequent monitoring of specific laboratory patterns, with particular attention during the titration phase and the recurrence of AHF episodes. In clinical practice, we recommend initiating SGLT2 inhibitors early during hospitalization, preferably before discharge, in patients admitted for AHF. This applies to those without symptomatic hypotension, the need for diuretic escalation, urinary tract infections, or a history of urinary disease. Treatment should also be avoided in patients who have received inotropic drugs or intravenous vasodilators within the past 6 h or have clear contraindications. If not initiated before discharge, treatment should ideally be started within 3 days afterward^70^ (*Figure* *2*). However, the therapeutic approach may differ between de novo HF and ADHF. In de novo HF, SGLT2 inhibitors should be initiated to counteract the underlying pathophysiological process and potentially restore baseline haemodynamic function. In ADHF, the decision should be guided by drug tolerability, prior HF treatment, the cause of hospitalization, patient history, and baseline renal function.

Patients' frailty, social, cognitive and educational level can increase the likelihood of wrong administration, discontinuation of medications both before and after hospitalization, which is associated with an increased risk of adverse outcome. In clinical practice many factors lead to under use or down‐titration of these compounds, including older age, hypotension, renal dysfunction, hyperkalaemia, cost, limited access, and/or clinician inertia.^71^, ^72^ Hospitalization provides a unique opportunity to optimize HF treatment in a monitored setting.^73^ Initiating SGLT2 inhibitors during this period enables better assessment of tolerability and side effects, improving the likelihood of achieving target doses in both short‐ and long‐term follow‐up.^74^, ^75^, ^76^ Strategies including a multidisciplinary guideline‐directed medical therapy (GDMT) team (pharmacist–physician) could improve HF therapeutic optimization during hospitalization and follow up.^77^

## 7 Conclusions

Early administration of SGLT2 inhibitors in‐hospital or shortly after discharge in AHF is safe and has shown clear prognostic benefits, although long term data are still lacking. Early initiation of SGLT2 inhibitors helps overcome therapeutic inertia, which is more likely to occur outside the hospital setting, particularly when patient management is led by non‐cardiology specialists. The simultaneous initiation of low‐dose quadruple medical therapy in patients hospitalized for heart failure should be encouraged. During the acute phase, early administration should be tailored based on haemodynamic status, congestion severity, and renal function. SGLT2 inhibitors have not demonstrated significant interactions with other HF therapies and are considered first‐line treatment in most cases due to their favourable safety profile and tolerability. The concurrent use of heart failure therapies may exacerbate the transient eGFR decline observed after early initiation, potentially contributing to therapeutic inertia in starting and up‐titrating these lifesaving treatments. In most cases, renal impairment is transient, with kidney function either returning to baseline or remaining stable over the long term. Conversely, when considering the extensive use of SGLT2 inhibitors during hospitalization for acute ischemic events, decisions should be guided by the presence of heart failure signs, the type and extent of myocardial damage, the timing and approach of the revascularization strategy, the risk of cardiogenic shock, and the patient's underlying metabolic status.

## Conflict of interest

Ruggero Mazzotta, Manuel Garofalo, Samuele Salvi, Matteo Orlandi, Gianluca Marcaccini, Pietro Susini, Luca Checchi, Alberto Palazzuoli, Carlo Di Mario, Maurizio Pieroni and Matteo Beltrami declare that they have no conflict of interest for this article.
